# Scalable Microreactor Concept for the Continuous Kolbe Electrolysis of Carboxylic Acids Using Aqueous Electrolyte

**DOI:** 10.1002/open.202200171

**Published:** 2022-10-06

**Authors:** Nils Baumgarten, Bastian J. M. Etzold, Juri Magomajew, Athanassios Ziogas

**Affiliations:** ^1^ Division Chemistry – Sustainable Chemical Syntheses Fraunhofer Institute for Microengineering and Microsystems IMM Carl-Zeiss-Straße 18–20 55129 Mainz Germany; ^2^ Technical University of Darmstadt Department of Chemistry Ernst-Berl-Institut für Technische und Makromolekulare Chemie Aalrich-Weiss-Straße 8 64287 Darmstadt Germany

## Abstract

The Kolbe electrolysis is a promising reaction to combine the usage of electrons as reagents and the application of renewable generated carboxylic acids as raw materials producing value added chemicals. Within this study, the electrolysis was conducted in a specially developed concept electrochemical microreactor and draws the particular attention to continuous operation and reuse of the aqueous electrolyte as well as of the dissolved unreacted feedstock. The electrolysis was conducted in alkaline aqueous solution using n‐octanoic acid as model substance. Platinized titanium as anode material in an undivided cell setup was shown to give Kolbe selectivity above 90 %. During the technically relevant conditions of current densities up to 0.6 A cm^−2^ and overall electrolysis times of up to 3 h, a high electrode stability was observed. Finally, a proof‐of‐concept continuous operation and the numbering up potential of the ECMR could be demonstrated.

## Graphical abstract

**Kolbe Electrolysis of n‐octanoic acid** in alkaline aqueous solution was conducted in flow, applying a bespoke electrochemical microreactor. Different parameters and reactor setups were studied, resulting in Kolbe selectivity of ≥90 % using an undivided cell setup with platinized titanium working electrodes. Besides, continuous operation with recycled electrolyte and reactor scaling was verified through concept studies.

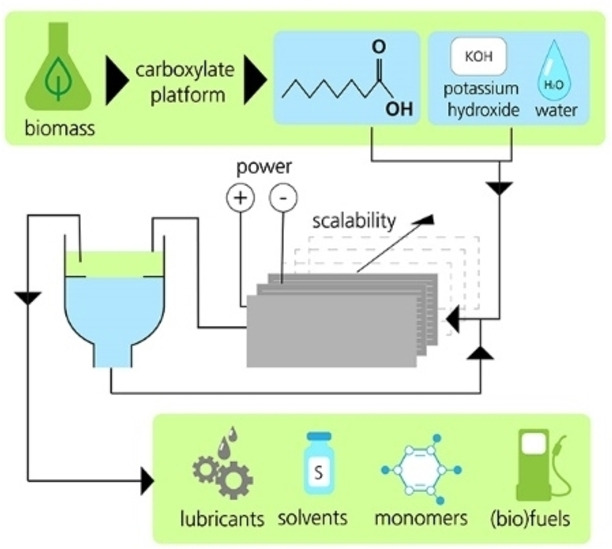

## Introduction

Electroorganic synthesis is attracting more and more attention especially due to the ongoing energy transition. The possibility to use “green” electrons, generated from renewable (energy) resources, as reagents to produce value‐added chemicals has given research in the field of electrochemistry a huge boost.^[1]^ Considering the 12 principles of green chemistry, electrochemical conversions allow the substitution of environmentally critical oxidants or catalysts with electrons, simplifying the separation step to isolate the product, thereby reducing waste.^[2]^

In addition, replacing fossil with renewable carbon sources constitutes an important step towards a more sustainable chemical production. Carboxylic acids are defined as important platform chemicals by the U. S. Department of Energy (DOE) and can be recovered from biomass.^[3]^ Besides the known biorefinery‐platforms, namely the sugar platform, where biomass is converted to C_5_ and C_6_ sugars with the help of enzymes, and the syngas platform, where synthesis gas (CO, H_2_ and CO_2_) is thermochemically produced from biomass, the carboxylate platform represents a third promising opportunity to recycle organic waste. Hydrolysis or fermentation is used to convert biomass to short‐chain carboxylic acids.^[4]^ Finally, those acids can be further converted to medium‐chain fatty acids (MCFA) in the same process with hydrogen and ethanol gained from second‐generation biomass by anaerobic bioconversions.^[5]^ MCFAs from biomass act as renewable feedstock for the production of biofuels or other value‐added products like fine chemicals, solvents, lubricants or monomers.^[6]^

**Scheme 1:**
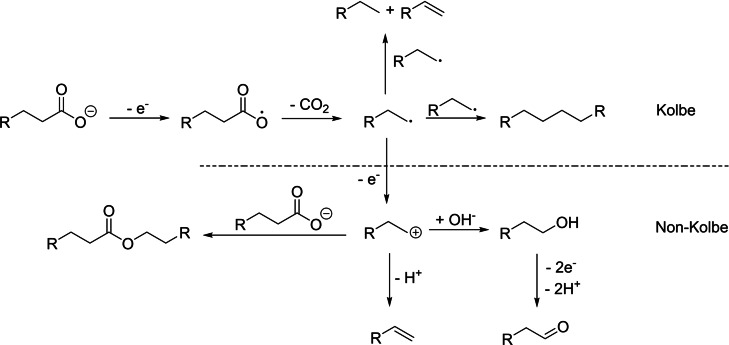
Mechanistic reaction scheme of Kolbe electrolysis.

Electrochemical conversion of fatty acids can be carried out via Kolbe electrolysis[^7^, ^8^] and the reaction scheme of the Kolbe process is depicted in Scheme 1. The carboxylic acid is deprotonated by the alkali hydroxide electrolyte added to the solution and the resulting carboxylate is oxidized at the anode. In the following step, an alkyl radical is formed via decarboxylation which can dimerize or disproportionate to alkanes or alkane and alkene, respectively (Kolbe).^[9]^ As a competitive process, oxidation of the alkyl radical can occur, where a carbenium ion is formed which can react with nucleophiles in consecutive reactions to form esters or alcohols (Most‐Hoefer reaction) that can be further oxidized to aldehydes or even carboxylic acids. Moreover, olefins can be produced via β‐H elimination (Non‐Kolbe).[^8^, ^10^, ^11^] Many studies have been carried out, focussing on the reaction conditions to tune the selectivities of the respective reaction pathways. Carboxylic acid concentration, pH value, current density, working electrode material, temperature and solvent were found to be important parameters to influence product selectivity. High acid concentrations (> 0.5 m) as well as high current densities (> 0.25 A cm^−2^) in combination with smooth platinum working electrodes are crucial to suppress the second oxidation step and favor the dimerization or disproportionation of alkyl radicals, where lower temperatures increase the rate of dimerization over the competing disproportionation.[^11^, ^12^, ^13^]

In general, Kolbe electrolysis is conducted in methanol with neutral to slightly acidic pH. However, from an environmental point of view, electrochemical synthesis in aqueous media is taken progressively more into consideration. Not only is it in accordance with one of the principles of green chemistry (rule 5),^[14]^ but the conductivity of, especially alkaline or acidic, aqueous solutions, is distinctly higher in comparison to organic solvents.^[15]^ Besides, biomass‐based carboxylic acids are recovered from the fermentation broth using an alkaline back‐extraction solution. Therefore, it would be beneficial to perform the electrolysis directly in the alkaline aqueous solution of fatty acids to combine both process steps as it is discussed in electrobiorefinery concepts. Urban et al. presented a proof‐of‐concept study combining the generation of carboxylic acids from biomass, alkaline extraction and direct Kolbe electrolysis of the extraction solution.^[16]^ While older literature stated that the selectivity for Kolbe products diminishes at higher pH values,[^12^, ^17^] more recent studies have shown that product selectivity is not significantly influenced by the pH.^[18]^ In addition, it was found that for using aqueous media, higher pH values can improve conversion of carboxylate, as the solubility of long‐chain carboxylic acids increases in alkaline media. As a consequence, the formation of electrode‐blocking agglomerates is suppressed.^[19]^ Furthermore, product separation can be simplified using an aqueous electrolyte. Products synthesized in the Kolbe process have lower polarities and densities than the aqueous solvent, which results in two separated liquid phases and allows for an easy and fast isolation of the organic product phase in a settler. In addition, phase separation allows a direct recycling of the aqueous phase in a continuous operation where reactant is constantly added.[^20^, ^21^, ^22^]

Considering such a continuous process, a proper electrochemical flow cell, for example realized by a thin gap between (microstructured) electrodes where the electrolyte is pumped through, is required. Flow cells have advantages over conventional batch cells, namely higher selectivity due to the defined residence time control, good heat transfer and high space‐time yields achieved by high surface‐to‐volume ratios and a lowered potential drop between the electrodes because of the diminished distance. Lower energy consumption or electrolysis with reduced amount of supporting electrolyte thus becomes feasible.^[23]^

Accordingly, Dos Santos et al. could improve the selectivity of the Kolbe electrolysis towards n‐octane from 51 % to 81 % using a flow cell where residence time was decreased to suppress the second oxidation step and the formation of a Hoefer‐Most product (1‐butanol).^[21]^ Wirth et al. studied trifluoromethylation of alkenes based on Kolbe electrolysis as well as the dimerization of 2‐phenylacetic and 2,2‐diphenylacetic acid in acetonitrile.^[24]^ Triethylamine was added as base neutralizing the electrolyte and suppressing the evolution of gaseous carbon dioxide. Electrochemical conversion of levulinic acid to 2,7‐octanedione and the non‐Kolbe electrolysis of monomethyl succinic acid to methyl acrylate in single‐pass and semi‐batch mode were studied in electrochemical flow cells.^[25]^ 3D printing techniques show a high potential for cost efficient and customizable manufacturing of electrochemical microreactors (ECMR) or flow cells that can be used to study the transformation from batch electrolysis into flow processes.

However, applied current densities, throughputs and residence as well as operation times range more towards lab scale dimensions. Enlarged reactor sizes with high surface to volume ratios and robust electrodes allowing to apply high current densities (above 100 mA cm^−2^) are rarely reported. In addition, there is a need for appropriate and flexible reactor concepts enabling the standardized operation on a laboratory scale considering parameter‐screening approaches and, at the same time, offering the versatility to number‐up electrochemical cells to address production or industrial scales.^[26]^ In contrast to batch upscaling where, for example, the dimensions of the reaction vessel are increased, leading to completely different mixing conditions and mass transfer properties, upscaling of electrochemical flow cells is mostly done by just numbering up the cells. The cell stack is then operated in parallel mode and the operation parameters can be adapted to each cell, obviating the need for renewed parameter screening or optimization experiments.^[27]^ Furthermore, electrochemical flow cell allow for a continuous operation of constant reactant feed and separation of the products. According to the aforementioned requirements, Ziogas et al. have developed and realized a novel flexible ECMR with microstructured electrodes with integrated heat exchanger, allowing defined temperature control to avoid hot spots in the microreactor due Joule reaction heat or high amperage, which is also the basis of this study.^[28]^

**Figure 1:**
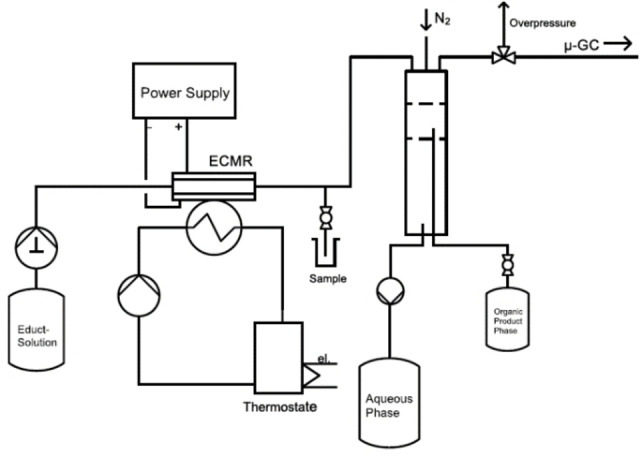
Flow chart of single‐pass Kolbe electrolysis in water.

Herein, we demonstrate a comprehensive study employing this microfluidic platform for a continuous Kolbe electrolysis of n‐octanoic acid in basic aqueous solution. Operation parameters, reactor setup and electrode materials were investigated in a single flow through arrangement (see Figure 1). In addition, long‐term stability of the ECMR was examined through performing longer electrolysis experiments. Based on this, a proof‐of‐concept continuous operation mode with recirculation of the electrolyte phase, addition of feedstock and withdrawal of product was realized. Finally, the possibility for easy numbering up was demonstrated and proved the scaling potential of the reactor concept.

## Results and Discussion

### Variation of Current Density

**Figure 2:**
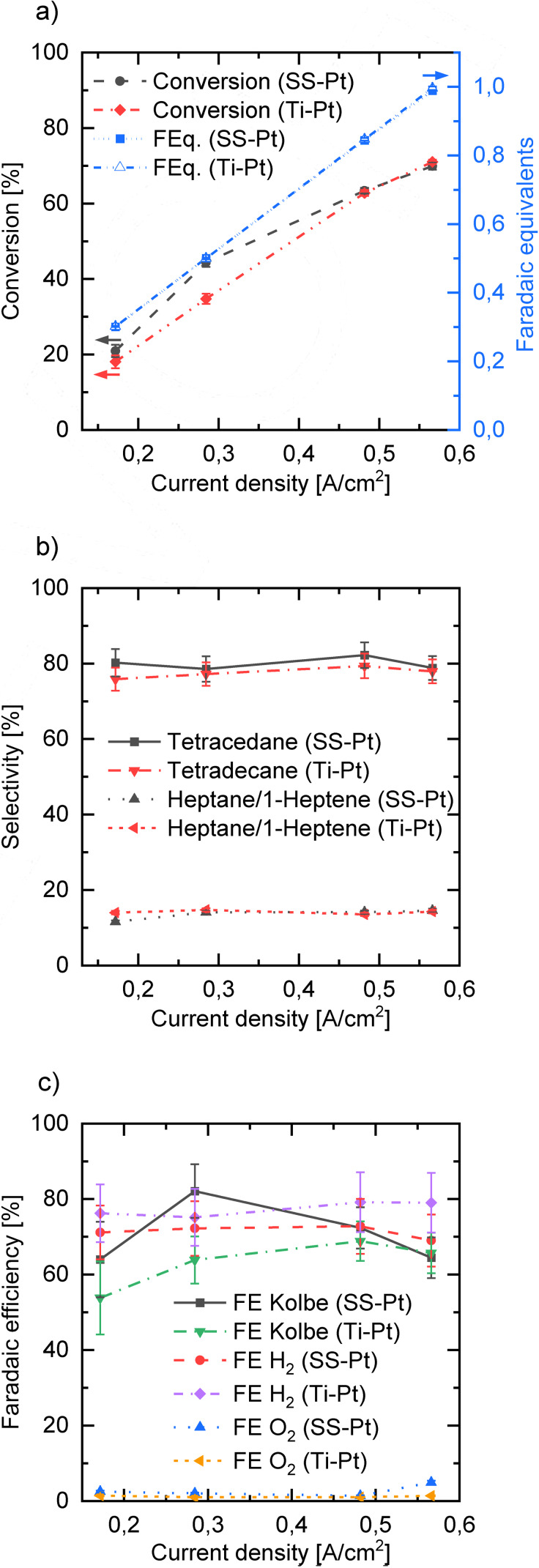
Conversion (left axis) and applied faradaic equivalents (right axis) (a), selectivity for Kolbe products tetradecane and heptane/1‐heptene (b) and anodic FE for all main Kolbe products (tetradecane, heptane, 1‐heptene), oxygen evolution reaction (OER, O_2_) and cathodic FE based on the hydrogen evolution reaction (HER, H_2_) (c) in relation to different current densities. In all electrolysis experiments, the residence time was set to 2.55 s (15 mL min^−1^ per cell). The concentration of n‐octanoic acid was 1.0 m and potassium hydroxide (1.5 m) was used as electrolyte. Electrolysis was performed in single‐pass mode with two cells in parallel using two different setups, namely platinum‐coated stainless steel as anode and nickel‐coated stainless steel as cathode (SS‐Pt||Ni‐SS) and platinum‐coated titanium as anode and platinum‐coated stainless steel as cathode (Ti‐Pt||Pt‐SS). Error bars result from standard deviation of sample measurements.

In order to identify optimal operation conditions for the Kolbe electrolysis within the ECMR, electrolyses at different parameters were carried out. At first, the current density was varied from 0.17 A cm^−2^ to 0.57 A cm^−2^ and the obtained results from the reaction mixture sample taken directly after the ECMR (see “Sample” in Figure 1) were compared. For the Kolbe process, the chemical as well as the electron selectivity are relevant performance indicators. The later one is the faradaic efficiency (FE), which can be given for the anode and for the cathode side. The former one is depicted in Figure 2b where the selectivity towards all detected Kolbe products in the organic phase based on n‐octanoic acid as reactant is summarized. Using platinized stainless steel as anode material, best results for the combined selectivity considering Kolbe products tetradecane, heptane and 1‐heptene (93.0 % and 94.1 %) were achieved for applied current densities of 0.28 A cm^−2^ and 0.48 A cm^−2^, respectively. Accordingly, the main side products detected, namely carboxylic esters and heptanal (see Figure S1, Supporting Information), showed the lowest selectivity for aforementioned current densities, whereas for lower as well as for higher current densities, a slightly increasing amount of side products and a decreasing amount of Kolbe products was found. According to the literature, the formation of the carbocations is increasingly favored at lower current densities (below 0.25 A cm^−2^) which leads to the formation of non‐Kolbe products.^[12]^ High current densities are known to favor the Kolbe reaction pathway. However, in our study, we could show that very high current densities in combination with moderate flow rates can also reduce the selectivity for the main Kolbe product (tetradecane). Due to high conversion, increasing amounts of gas bubbles are formed, covering parts of the electrode surface and so causing current density instabilities at the wetted electrode areas. Consequently, over‐oxidation to, for example, carbocations can occur and the Kolbe selectivity is reduced. Hence, at very high current densities, higher flow rates should be applied, increasing the mass transfer and improving the removal of gas bubbles to maintain high Kolbe selectivity. In addition, it was also shown that high hydroxide ion concentrations (high pH value) do not necessarily implicate in the formation of non‐Kolbe products like alcohols (aldehydes) or esters, which has been also shown in one of our previous works^[22]^ and had been studied in detail by Harnisch et al.^[18]^ Despite the discussed results, we could reach a very high selectivity for Kolbe products (>91 %) in the investigated current density range, and the detected differences where only small.

Conversion can also be discussed in relation to different applied current densities (Figure 2a). Due to the steady flow rate at all four current density values, increasing amounts of charge (faradaic equivalents (FEq.)) are applied within the same residence time. In line with our expectations, higher current densities lead to higher conversion of carboxylic acid. Considering the first three data points, the conversion grows almost linearly with the current density. Only for the last data point, the rise in conversion (63.4 % to 69.9 %) is smaller, which could mean that conversion per residence time approaches a limiting value near 1.0 FEq for the investigated flow rate. Another reason might lie in the higher amount of gas formed at higher current densities. In general, there are 1.5 mol of gas per converted mol of carboxylic acid, namely carbon dioxide (CO_2_), deriving from the decarboxylation of the oxidized carboxylate and hydrogen (H_2_), which originates from the counter reaction. Thus, the increasing gas amounts could block the access of electrolyte to the electrode surface.

Depending on the selectivity and carboxylic acid conversion, the FE for all detected products was calculated, where the highest FE for Kolbe products (82.1 %) was achieved at a current density of 0.28 A cm^−2^ (Figure 2c). Lower values were found for 0.17 A cm^−2^ and 0.57 A cm^−2^, revealing 64 % and 64.5 % FE, respectively. This may be due to the high conversion of carboxylic acid, which can build a blocking layer at the electrode surface suppressing solvent oxidation.^[29]^ Another reason could be the loss of electrode selectivity due to increased cell voltage (5.2 V at 0.57 A cm^−2^) and oxygen evolution taking place at the anode.

Furthermore, the FE for the cathodic hydrogen evolution reaction (HER) was calculated according to the amount of H_2_ detected by a mass flow meter and micro‐gas‐chromatograph (micro‐GC) and the applied charge. Almost the same FE (72 %–69 %) resulted for H_2_ at the different current densities which is in accordance with the overall anodic FE where all detected products (main Kolbe products and side products, for example, heptanal, ester, oxygen) have been taken into account for the calculation.

However, not all applied charge could be directly dedicated to electrochemical processes, and different sources of the detected charge loss occurring during the process have to be discussed. Due to the undivided cell setup, H_2_ which is formed at the cathode side as counter reaction (HER), could be partly oxidized to protons at the anode again before the gas molecules can leave the electrochemical cell (redox‐shuttle). This aspect is further discussed below, when undivided and divided cell setups are compared. In addition, as the pH is not only influenced through the generation of protons, but also from the organic compounds and the reaction of formed carbon dioxide with hydroxide‐ions a proton balance cannot be easily made from the pH and charges going to hydrogen oxidation reaction (HOR) cannot be given and are out of scope of this study.

### Variation of Residence Time and System Pressure

**Figure 3:**
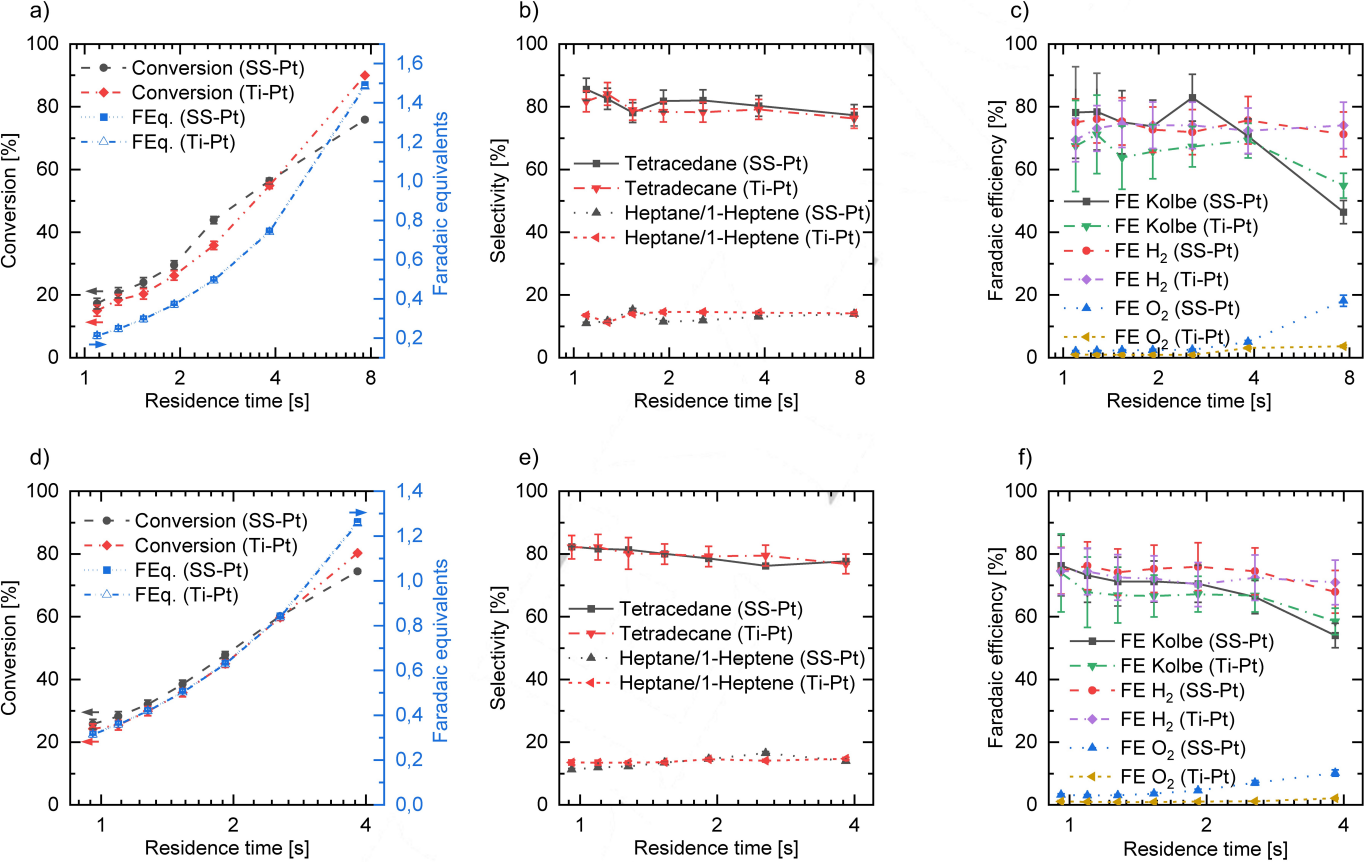
Flow rate variation at constant current densities of 0.28 A cm^−2^ (a–c) and 0.48 A cm^−2^ (d–f). Electrolysis was performed in single‐pass mode with two cells in parallel using two different setups, namely platinum‐coated stainless steel as anode and nickel‐coated stainless steel as cathode (SS‐Pt||Ni‐SS) and platinum‐coated titanium as anode and platinum‐coated stainless steel as cathode (Ti‐Pt||Pt‐SS). Concentration of n‐octanoic acid was 1.0 m and potassium hydroxide (1.5 m) was used as electrolyte. Graphs show conversion (left axis) and applied faradaic equivalents (right axis) (a,d), selectivity for Kolbe products tetradecane and heptane/1‐heptene (b,e) and anodic FE for all main Kolbe products (tetradecane, heptane, 1‐heptene), oxygen evolution reaction (OER, O_2_) and cathodic FE based on the hydrogen evolution reaction (HER, H_2_) (c,f). Error bars result from standard deviation of sample measurements.

In addition to current density variation, the influence of different residence times on the reaction performance was evaluated by changing the flow rate from 5 mL min^−1^ to 40 mL min^−1^ per cell. According to the best achieved results for the FE in the current density variation, 0.28 A cm^−2^ and 0.48 A cm^−2^ were chosen as current densities to study the effect of the residence time. As expected, in both cases conversion is increasing for increasing residence times due to the fact that more FEq. could be applied. Considering chemical selectivity for Kolbe products, the best values were achieved for the lowest residence times (96.5 % for 0.28 A cm^−2^ and 93.6 % for 0.48 A cm^−2^), showing a trend towards reduced selectivity for Kolbe products at higher residence times (Figures 3b and 3e). Because of a weakened mass transfer present at slow flow rates (high residence times), the second oxidation step for the adsorbed alkyl radicals becomes more likely, resulting in an alkyl cation which reacts to alcohols, esters or olefins. This is especially seen by an increasing amount of heptanal (Figure S1). However, even at high residence times, good selectivity for Kolbe products (91.3 % at 7.66 s (0.28 A cm^−2^) and 91.6 % at 3.83 s (0.48 A cm^−2^)) could be reached. Hence, the variation of residence time also confirms that, within the studied residence time and current density range, selectivity is mainly driven by starting concentration and electrode material. Although conversion per residence time is increasing for slow flow rates without a significantly negative impact on the selectivity of the desired products, the productivity is decreasing for both current densities. For the lowest residence time, productivity was found to be 0.39 mol h^−1^ (1.09 s) and 0.65 mol h^−1^ (0.96 s), respectively, decreasing in both cases for higher residence times. Only for 0.28 A cm^−2^, a local maximum of 0.42 mol h^−1^ was achieved at 2.55 s residence time (see Supporting Information). Furthermore, lower cell voltages were reached, which can be attributed to an improved mass transfer induced by the reduced diffusion layer at the electrode surface.^[30]^ Hence, the trend in productivity is coincident with the tendency of lower energy consumption for higher flow rates.

With respect to the anodic faradaic efficiency (Figures 3c and 3f), a similar trend was found, namely lower residence times favoring electron selectivity for the formation of Kolbe products (78.2 % at 0.28 A cm^−2^, 76.3 % at 0.48 A cm^−2^), where a local maximum of 82.9 % was found at residence time of 2.55 s (0.28 A cm^−2^). Long residence times led to a decrease in faradaic efficiency considering the Kolbe reaction as well as the overall anodic FE. The formation of heptanal, originating from the alcohol formation via the non‐Kolbe pathway and a consecutive oxidation to form the aldehyde, and oxygen, as a competitive anode reaction, are consuming increasing amounts of charge for slower flow rates. In case of the oxygen evolution reaction (OER), the carboxylate layer on the plane platinum electrodes, generally preventing solvent oxidation, degrades due to high acid conversion, and oxidation of the solvent is consequently less hindered. In addition, mass transfer (of carboxylic acid) at low flow rates is reduced, regeneration of the carboxylate layer is decreased and OER can occur at (carboxylate‐)free electrode sites. For a continuously operated electrochemical flow process, where the electrolyte is recycled and the unreacted substrate is still dissolved in the recycled aqueous phase, it can in conclusion be more effective to reduce the conversion per cycle as selectivity and faradaic efficiency are increased.

**Figure 4:**
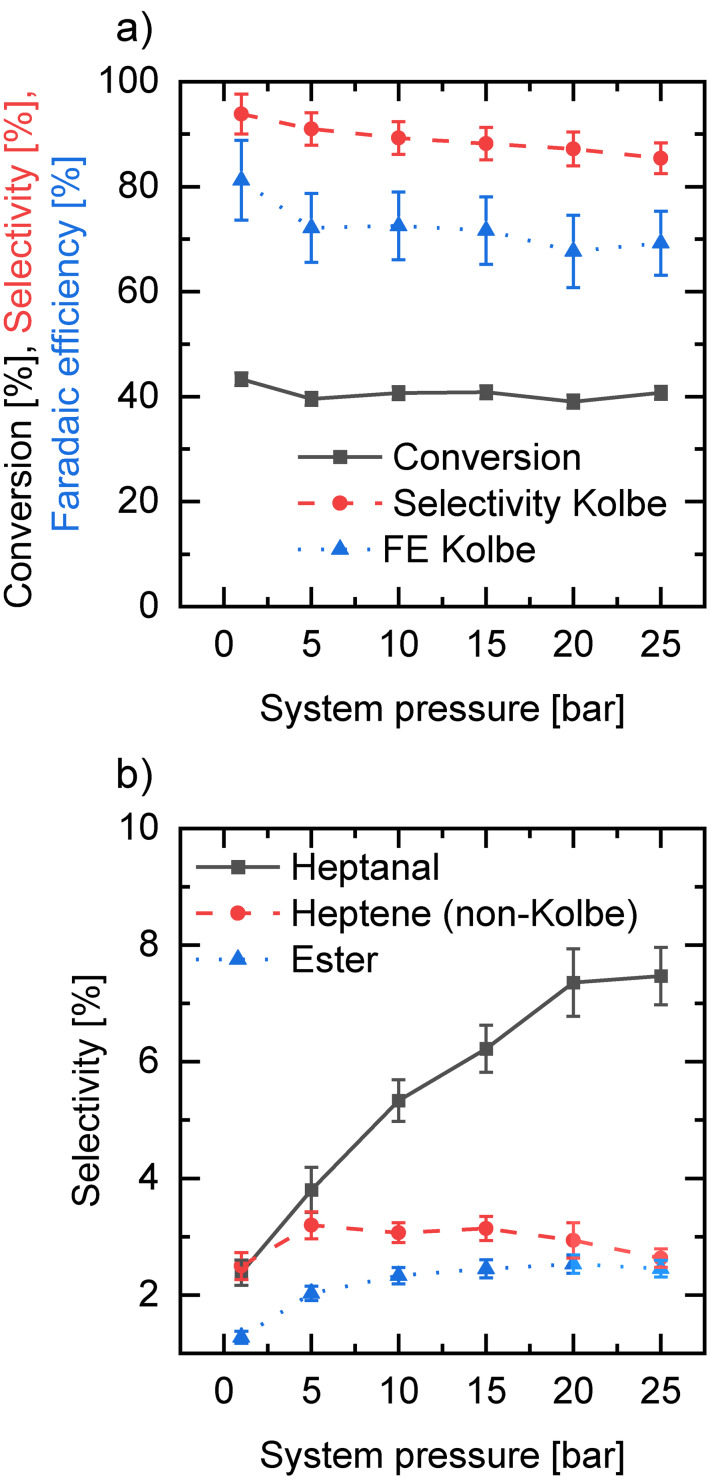
a) Selectivity and FE for main Kolbe products tetradecane, heptane and 1‐heptene and conversion depending on different system pressures. b) Selectivity for non‐Kolbe products (heptanal, ester, heptene (non‐Kolbe)) relative to increased system pressure. Error bars result from standard deviation of sample measurements.

In order to study the influence of increased system pressure, a needle valve was used to control the backpressure after the ECMR. The normal system pressure with two cells in parallel and overall flow rate of 30 mL min^−1^ (15 mL min^−1^ per cell, 2.55 s residence time) and applied current density of 0.28 A cm^−2^ amounted to 1.1–1.2 bar absolute pressure. A conversion of carboxylic acid per residence time of 43 % with a chemical selectivity for Kolbe products of 93.8 % and FE of 81.2 %, was achieved. All relevant results were found to decrease with increasing system pressure. Conversion descends to 39–40 % and FE to 72 % for main Kolbe products when the system pressure was increased to 5 bar. Furthermore, chemical selectivity for tetradecane, heptane and 1‐heptene was also reduced to 91 %. This effect was also reported by Kappe et al. who had studied the influence of backpressure on the anodic methoxylation of *N*‐formylpiperidine using their developed “pipe cell” design electrochemical flow reactor.^[31]^ While further increasing the system pressure leaves the conversion constant at 39–40 %, the selectivity as well as the FE for Kolbe products show a decreasing trend. This is in accordance with the selectivity of the main side products (i. e., heptanal, non‐Kolbe olefins and octanoic acid heptyl‐ester) which are increasing for higher pressures (Figure 4). The basic intention in reduction of system pressure is to minimize the negative effect of the gas evolving during the reaction in the electrochemical cell. In fact, gas bubbles do not contribute to the conductivity of the system, therefore increasing the Ohmic drop and (artificially) reducing the active electrode area which can lead to unequal current density distribution along the electrode.^[32]^ Higher system pressure can increase the solubility of gas molecules in the electrolyte and compress the gas segments, reducing the amount of gas bubbles in the electrochemical cell. As most of the formed carbon dioxide immediately reacts with hydroxide ions to carbonate in the liquid phase, bubble formation in the microchannels is mainly caused by hydrogen. The solubility of H_2_ in aqueous potassium hydroxide is approximately 0.4 ⋅ 10^−6^ mol mL^−1^ bar^−1^.^[33]^

Considering a flow rate of 15 mL min^−1^ per cell and a conversion of 40 %, there would be 3 mmol min^−1^ of H_2_ formed in the ECMR where only 0.2 % are soluble at normal back pressure. Increasing the pressure to 25 bar and assuming the same conversion, only 5 % of the formed hydrogen would dissolve. Thus, the decrease of gas segments due to compression is more decisive than the solubility of gas molecules in the electrolyte. In fact, by increasing the pressure from 1.1–1.2 bar to 5 bar, a decrease of cell voltage from 4.5 V to 4.2–4.3 V at constant cell current was achieved, confirming that gas segments were compressed. However, the overall cell performance, as shown in Figure 4, is reduced. A possible explanation for this effect could be that despite the compression of gas segments in the electrolyte, which reduces the cell voltage, desorption of H_2_ gas molecules from the cathode surface is hindered at increased back pressure. The impeded cathodic process also effects the anodic conversion of carboxylic acid. Furthermore, adsorbed gas bubbles at the electrode surface lead to a more inhomogeneous current density distribution which can cause overoxidation of the substrate. Accordingly, increasing amounts of non‐Kolbe products were found at elevated back pressures.

### Variation of Cell Setup (Divided and Undivided Cell)

To discuss the influence of a divided cell setup, electrolysis was also carried out using an anion exchange membrane (AEM) between two microstructured electrodes. Table 1 compares the achieved results for undivided and divided cell. With regard to chemical selectivity, the same selectivity for Kolbe products (undivided: 92.7 %, divided: 92.7 %) was found for both cell setups, whereas the FE differed. The comparable selectivity can be explained due to the fact that platinum was used as anode material in both cases. The lower FE results as the degree of conversion decreased from 44 % (undivided) to 28 % (divided), despite the same applied current density. A possible reason for the reduced acid conversion could originate from strong local pH changes. In most electrochemical reactions, protons (or acidic molecules) are formed at the anode, whereas the cathode reaction generates bases. In that case, the counter reaction can compensate the pH change at the working electrode, ensuring a stable conversion which could be negatively affected when the pH value is changing distinctly.^[34]^ Using a divided cell setup, this counterbalance is not given as ion transport through the membrane is too slow and a noticeable pH shift in the anodic chamber can result. Considering the Kolbe reaction, acidic CO_2_ is formed, which consumes hydroxide ions according to the aforementioned reaction, thus shifting the pH to lower values in the anodic chamber. In an undivided cell setup, hydroxide ions which are generated at the cathode during HER can compensate this pH shift. Using an AEM to separate the electrode chambers, diffusion of hydroxide ions is reduced due to increased transport resistance through the membrane. Accordingly, this compensatory mechanism is diminished in a divided cell setup and the local pH shifts to lower values, which in turn may lead to local decreased solubility of carboxylic acids in water, resulting in reduced conversion. In fact, the pH value of the aqueous phase after electrolysis in a divided cell setup was 10.2 at 25 % conversion, whereas measured pH values for samples originating from a residence time variation with comparable conversions were 13.0 (24 % conversion), 11.9 (28 %) and 10.6 (36 %).

**Table 1:** Comparison of selectivity for main Kolbe products tetradecane, heptane/1‐heptene and conversion depending on different cell setups as well as FE for all three Kolbe products, OER and HER. Electrolysis was conducted with 15 mL min^−1^ (2.55 s residence time) per cell and 0.28 A cm^−2^. Concentration of n‐octanoic acid and KOH was 1.0 m and 1.5 m, respectively. For the divided cell setup, a 0.5 m KOH solution was used as catholyte. Error bars result from standard deviation of sample measurements.

Parameter	Undivided Cell	Divided Cell
Conversion [%]	44±1	28±2
Selectivity Tetradecane [%]	79±3	79±3
Selectivity Heptane/1‐Heptene [%]	14.1±0.4	14.0±0.4
FE Kolbe Products [%]	82±7	52±6
FE OER [%]	2.1±0.2	5.8±0.6
FE HER [%]	72±7	57±6

Accordingly, the aqueous phase pH value was significantly lower for the electrolysis conducted in a divided cell setup having reached a conversion of only 25 %. Harnisch et al. discussed the impact of the pH value on the solubility of n‐octanoic acid agglomerates in water in the bulk and locally at the electrode surface and concluded that, for lower pH values (close to the p*K*_a_ of the carboxylic acid), agglomerates where formed. These can block the electrode surface or hinder charge transfer, thus diminishing the overall performance of the electrolysis which could explain the detected effect in the cell setup variation.^[19]^ Moreover, in the undivided setup, the ratio of gaseous H_2_/CO_2_ is increasing, implying a reduced amount of CO_2_ leaving the cell in gaseous form due to the reaction with hydroxide ions. This effect can be reduced in an undivided cell setup where H_2_ can partly expel more gaseous CO_2_ before its reaction with hydroxide ions. Finally, results for overall FE for anode and cathode processes could not be increased using the divided cell setup, which is a strong indication that a redox shuttle is not responsible for FE losses in an undivided cell setup. Considering the discussed results, it can be concluded that an undivided cell setup is favored for the reaction.

### Stability Consideration of Different Electrode Materials

Since the ECMR is intended to be used in a continuous process, the long‐term stability of the electrode material has to be taken into account. When different residence times were studied, the color of the aqueous phase after phase separation showed a trend from colorless over light yellow to a light orange color for higher residence times (see Supporting Information). Furthermore, a fine red‐brown precipitate sedimented in the aqueous phase of the samples after several hours. The precipitate was isolated and analyzed via energy‐dispersive X‐ray (EDX) measurements (see Supporting Information). Iron and oxygen amounted for most of the detected elements and might originate from iron oxide that was generated during the electrolysis. It can be assumed that this metal oxide comes from corrosion processes at the 3D‐printed anode were stainless steel was the bulk material. Although all major electroactive parts were either electroplated with platinum or coated with insulating PTFE, the platinum layer at the edges of the microchannels could reveal minimal parts of uncovered stainless steel due to mechanical stress. In addition, there could be a small area of stainless steel between the inlet of the electrolyte and the tubing connections which might be exposed to the electrolyte. As a consequence, anodic corrosion of stainless steel can be assumed to contribute to charge loss during the reaction especially when high current densities in combination with increased residence times are applied. Overall, the anodic FE decreases from approximately 90 % to 77 % when the residence time was increased from 1 to 4 s which is consistent with the color change of the aqueous reaction mixture.

To address this effect, another anode material was tested. Due to the corrosive effects of the anode base material (stainless steel), titanium (Ti) was used as alternative anode base material which was electroplated with Pt to ensure the selectivity of the process. Titanium shows good corrosion stability as thin oxide layers are formed immediately when Ti is anodized. Those oxide layers passivate the surface and prevent further oxidation of the material.^[35]^ Accordingly, the microstructured electrodes with Pt were used as cathode in that case (Ti‐Pt||Pt‐SS). To compare the new electrode material with the original setup (SS‐Pt||Ni‐SS), the same current density range and flow rates were investigated and chemical as well as electron selectivity is depicted in Figure 2 and Figure 3.

With respect to the selectivity for the main Kolbe products (≥90 %), comparable results were found with a tendency towards slightly better results for the SS‐Pt anode. For high current densities, the same conversion per residence time was achieved, whereas differences could be detected for lower current densities. Accordingly, better FE for the Kolbe products was realized for 0.17 A cm^−2^ and 0.28 A cm^−2^. When higher current densities were applied, comparable results were obtained.

The aforementioned effect can be also seen in the flow rate variation, where slightly higher conversions for SS‐Pt result in a better FE for the Kolbe products for both investigated current densities. At higher residence times, the performance of the SS‐Pt setup is decreasing and better results for FE were achieved for the Ti‐Pt anode setup. This effect can be explained by the FE for the OER which is significantly increasing for higher residence times using the SS‐Pt anode, whereas only a small increase was detected for Ti‐Pt (Figure S2).

As stated before, due to mechanical stress or insufficient coating, parent material of the electrode (SS or Ti) can be exposed and comes in contact with the electrolyte. In case of Ti as parent electrode material, a passivating oxide layer is formed which can lead to an increased resistance (higher cell voltage) for long term applications, however no further oxidation or electrode degradation will occur. In contrast, stainless steel, when exposed to the electrolyte, does not form any passivating layer, but is continuously corroded and facilitates the OER, which can be seen by the growing amount of detected oxygen and the iron oxide precipitate in the aqueous phase for high residence times. No precipitation or color change of the aqueous phase was detected when Ti was used as parent material for the anode.

In summary, platinized titanium shows comparable results to platinized stainless steel considering chemical selectivity and conversion of carboxylic acid. Hence, Ti as anode base material shows higher stability and resistance to corrosion especially for harsh conditions (high current densities in combination with high residence time) and longer electrolysis times as discussed in the following section.

### Proof‐of‐Concept Continuous Operation Mode and Numbering Up Approach

In order to show a proof of concept for the continuous operation with recycled electrolyte, the electrolysis was conducted for one hour in single‐pass mode. After the phase separation, the aqueous phase containing unreacted acid was reconcentrated with carboxylic acid to 1.0 m and the electrolysis was repeated. Three cycles have been carried out for each setup (SS‐Pt||Ni‐SS and Ti‐Pt||Pt‐SS) and the results after one hour electrolysis are shown in Table 2 and Table 3. In all cycles, no unconverted octanoic acid was detected in the organic phase after GC‐MS/FID analysis which proves that the spontaneous phase separation is sufficient and a recycling can be realized, as no unconverted starting material is lost in the organic product phase which is removed from the system. Considering conversion and selectivity, comparable results were achieved with both setups where, especially in the first two cycles, a slightly better conversion was found for the SS‐Pt||Ni‐SS setup. Chemical selectivity for the main Kolbe products is almost identical which shows that the Pt coating of both working electrode base materials (stainless steel and titanium) is sufficient to direct the reaction pathway. Small detected differences in conversion for the first two cycles could be assumed to originate from different activities for the HER depending on the used cathode material. However, in both cases, conversion was significantly decreasing in the third cycle which could be accounted for either by an accumulating contamination or impurity in the reused electrolyte solution or an electrode effect, where the latter one is most probable. Inspection of the electrodes after each cycle and especially after the third cycle revealed significant differences.

**Table 2:** Results of indirect recirculation using recycled electrolyte operating with two cells in parallel (SS‐Pt||Ni‐SS, 0.48 A cm^−2^, 2.55 s residence). Error bars result from standard deviation of sample measurements.

Cycle^[a]^	Conversion [%]	Selectivity Tetradecane [%]	Selectivity Heptane/1‐Heptene [%]	FE Kolbe [%]
1	64.6±0.8	81±3	12.4±0.4	72±6
2	65.4±0.7	82±3	12.3±0.4	72±5
3	56.3±0.9	78±3	13.4±0.4	64±5

[a] Results were taken from the T piece after the ECMR after one hour of single‐pass electrolysis. For the second and third cycle, 1.5 L of aqueous reaction mixture were taken and carboxylic acid was added to reach a concentration of 1.0 m. KOH was added according to the amount of fresh carboxylic acid in a ratio of 1 : 1.5 (acid:KOH) to ensure sufficient solubility. Error bars result from standard deviation of sample measurements.

**Table 3:** Results of indirect recirculation using recycled electrolyte operating with two cells in parallel (Ti‐Pt||Pt‐SS, 0.48 A cm^−2^, 2.55 s residence). Error bars result from standard deviation of sample measurements.

Cycle^[a]^	Conversion [%]	Selectivity Tetradecane [%]	Selectivity Heptane/1‐Heptene [%]	FE Kolbe [%]
1	62.2±0.8	81±3	12.3±0.4	69±5
2	61.8±0.8	82±3	10.6±0.3	69±5
3	57.8±0.9	78±3	13.7±0.5	62±5

[a] Results were taken from the T piece after the ECMR after one hour of single‐pass electrolysis. For the second and third cycle, 1.5 L of aqueous reaction mixture were taken and carboxylic acid was added to reach a concentration of 1.0 m. KOH was added according to the amount of fresh carboxylic acid in a ratio of 1 : 1.5 (acid:KOH) to ensure sufficient solubility.

A dark black and dark red residue was deposited on the Ni‐SS cathodes when high residence times were applied. Furthermore, the color of the aqueous solution turned from light yellow to light red over the course of the three cycles. Accordingly, it can be concluded that corrosion of exposed stainless steel is occurring also for long electrolysis times. Apart from fine residue being flushed out of the electrochemical cell, thus coloring the reaction mixture, some precipitate is deposited on the counter electrode, reducing the active electrode area which hinders the counter reaction (HER). In addition, the dark parts on the Ni cathode could originate from the formation of nickel hydride which is a known phenomenon in alkaline water electrolysis and can reduce the catalytic activity of nickel concerning the HER.^[36]^ Using the Ti‐Pt||Pt‐SS setup, no visible electrode effects could be detected. This might also explain the decrease in conversion for Ti‐Pt anode material of only 6.5 % compared to a 13.5 % decrease using the SS‐Pt||Ni‐SS setup. Nevertheless, a reduced conversion was detectable in both cases which may be caused by the activity loss of the electrodes over the three hours of combined reaction time. Furthermore, based on the recycling concept, the concentrations of potassium and carbonate are growing over the cycles where only that of carbonate is detectable by Raman spectroscopy. The carbonate content in the aqueous phase after the electrolysis is increasing from approximately 0.4 m over 0.6 m to 0.75 m for both setups within the three cycles due to the reaction of carbon dioxide and hydroxide ions. However, no evidence was found that the growing concentration of carbonate is accountable for the lower conversion. At this point, we can only speculate that this is rather improbable as a decreasing conversion should then already result for the second cycle. Another aspect concerning electrolyte change is the pH value of the starting solution which decreased from 13.5–13 to 10.5 for the last cycle. Accordingly, lower solubility of n‐octanoic acid and different agglomerates of carboxylic acid or carboxylate near the electrodes could be a consequence, in turn reducing conversion. Those findings have to be further investigated in direct recirculation modes to clarify the influence of changing pH value on the performance of the reaction.

**Figure 5:**
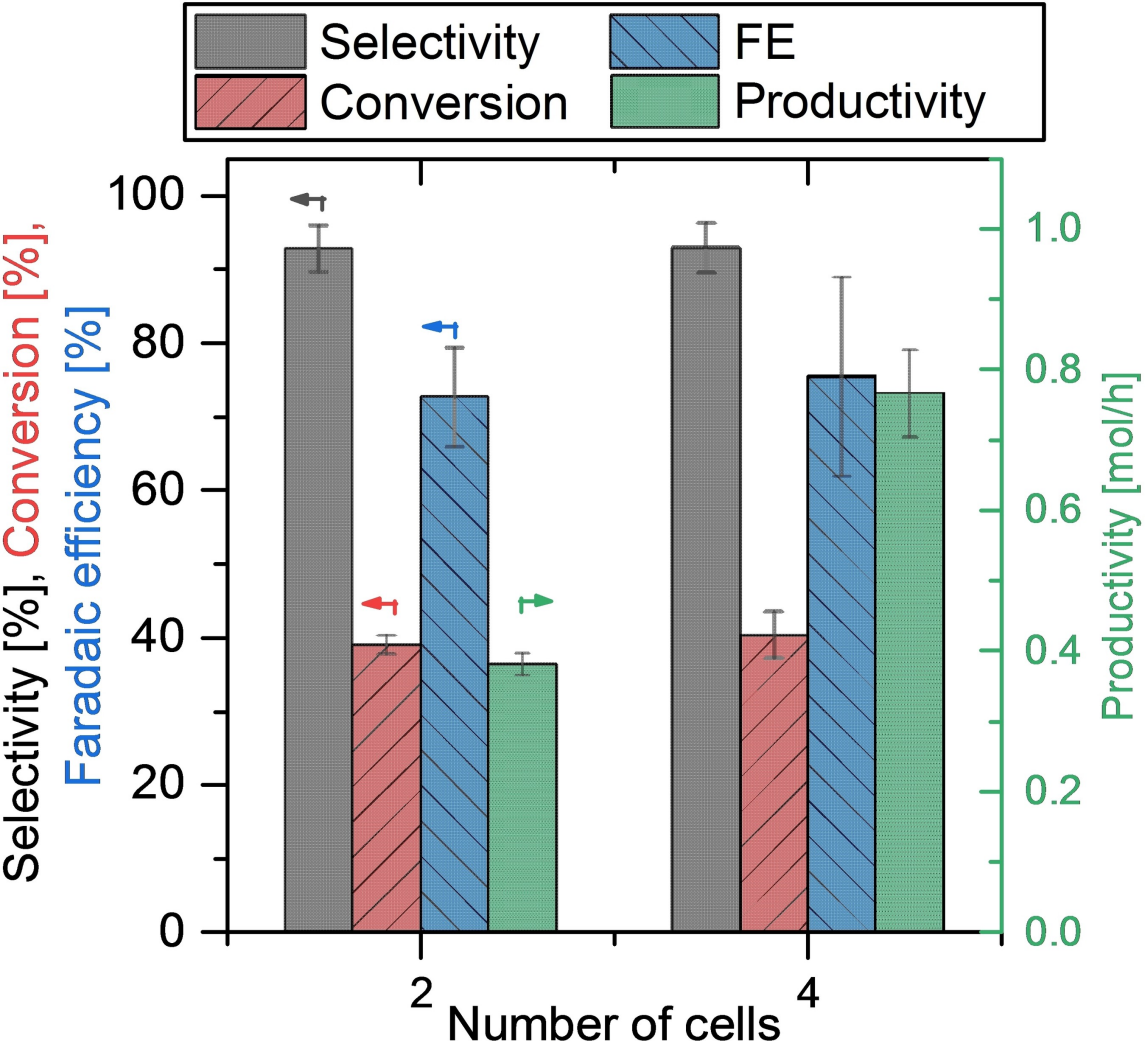
Productivity (green bars, right scale) as well as conversion, selectivity and FE for Kolbe products tetradecane, heptane, 1‐heptene (left scale) in relation to the number of electrochemical cells operated in parallel. Error bars result from standard deviation of sample measurements.

To demonstrate the scaling potential of the ECMR, the electrochemical synthesis was conducted with two and with four cells operated in parallel by increasing the stack size. In both experiments, the same current density (0.28 A cm^−2^) and residence time (2.55 s) was applied to each cell. The achieved results are depicted in Figure 5, where very similar values were reached for conversion, FE and selectivity for the Kolbe products tetradecane, heptane and 1‐heptene. As intended, productivity could be doubled from 0.38 mol h^−1^ to 0.77 mol h^−1^ by duplicating the number of cells, which prooves the fast and easy scaling potential of our ECMR.

## Conclusion

In this work, a microfluidic electrochemical cell was successfully employed to carry out the Kolbe electrolysis of n‐octanoic acid in an aqueous electrolyte. In single‐pass mode, the influence of current density, residence time, pressure and cell setup on the performance of the electrolysis was studied. A current density between 0.3–0.5 A cm^−2^ and residence times ≤2.5 s without any backpressure were identified as optimal conditions for single‐pass electrolysis. It was confirmed that the selectivity for Kolbe products is predominantly driven by the working electrode material, which should be a smooth platinum surface, and a high concentration of carboxylic acid. Performance and selectivity of the reaction in flow can be further improved by applying high current densities and low residence times which, in addition, increase productivity and lower energy consumption for the production of Kolbe products, factors becoming exceedingly relevant considering a continuous process. An undivided cell setup gave better results concerning Kolbe selectivity and conversion per residence time compared to a cell divided by an anion exchange membrane. Most likely, the locally changing pH in a divided cell is responsible for the reduced conversion of carboxylic acids. Moreover, the long term stability of the electrode materials, especially when applying demanding conditions and long electrolysis times, has shown to be a crucial factor for the application of the ECMR in a continuous process. The developed platinized titanium anodes showed comparable results to the original platinized stainless steel electrodes, while the former reveal a better long term stability and no visible corrosion effects when high current densities in combination with high residence times were applied. Insufficiently coated parts, bringing electrolyte into contact with the electrode base material, show passivation in case of titanium, whereas corrosion effects and the formation of solid iron oxides have been observed for stainless steel. A proof of concept considering the scaling potential of the reactor concept was demonstrated, where productivity was increased depending on the number of electrochemical cells operated in parallel. Furthermore the aqueous reaction mixture was recycled and reused in order to simulate a continuous operation. A decrease in conversion was found after three hours of reaction time, and the combination of reduced electrode activity and a change in pH value can be assumed to account for the observed effects. Again, platinized titanium showed only a minor performance reduction in comparison to stainless steel where visible corrosion effects were prominent. In a long‐term and directly recirculated electrolyte solution, the impact of the adjusting pH value must be further investigated to implement a continuous process.

## Experimental Section

### Chemicals

Chemicals were purchased from PanReac AppliChem, Carl Roth and Scharlau and used without any further purification if not stated otherwise. Deionized water was freshly produced using an ultra‐pure water system (Millipore, Milli‐Q Plus, Merck).

### Electrochemical Setup and Electrolysis

An electrochemical microreactor was used for the electrolysis. The electrodes were fabricated by selective laser melting (stainless steel), coated with polytetrafluoroethylene (PTFE) for insulation, microstructured with a side‐milling cutter and electroplated with platinum. A microstructured reactor plate provides an active electrode area of 42.6 cm^2^. For manufacturing details, see our previous publication.^[28]^ A plane stainless steel plate, coated with Nickel (∼5 μm thickness), was used as counter electrode. Both electrodes were assembled in a reactor housing and pressed together with PTFE‐sealing (Chemraz®) in between to prevent leakage. The PTFE‐coated bars defining the mirochannels of the electrode, (dimensions of channel: 100×0.76×0.15 mm^3^, 56 microchannels per electrode side) act as spacers between the electrodes and provide a reaction volume of 0.64 mL per undivided cell. For a divided cell setup, two microstructured electrodes were assembled with an anion exchange membrane (AEM) in between, which was purchased from Fumatech GmbH (FUMASEP® FAA‐3‐50, 50 μm thickness, bromide as counter ion). For an additional setup variation, the microstructured platinized electrode was used as counter electrode and a plane platinized titanium electrode was used as anode material (platinum layer thickness: ∼5 μm).

The aqueous electrolyte solution (KOH: 1.5 m, n‐octanoic acid: 1.0 m) was pumped through the cell using a preparative HPLC pump (Knauer WellChrom k‐1800). A Genesys GEN10‐500 from TDK‐Lambda and a HCS‐3600 from Maas‐Electronic were used as power supplies and operated in constant current mode. The heat exchange medium (Isopropanol) was pumped through the integrated heat exchanger of the microstructured electrodes with a thermostat (CC3, Huber; temperature=20 °C).

After the reaction mixture has left the electrolysis cell, it is collected in a settler where the separation of the three phases (gas, organic phase, aqueous phase) takes place. The amount of gas is detected via a mass‐flow‐meter Smart‐Trak 100 (Sierra Instruments) and afterwards analyzed via a micro‐gas‐chromatograph. The lower aqueous phase, which contains only minor parts of organic residues, is continuously removed from the settler in a reservoir. Quantification is done via Raman‐spectroscopy after complete phase separation. The entire organic phase is collected after the electrolysis has finished and analyzed via a gas chromatograph (GC) with mass selective detector (MS) and flame ionization detector (FID). Samples during the synthesis were taken via a T piece after the reactor and analyzed as stated above after phase separation.

### Chemical Analysis

The gas flow was detected with a thermal mass‐flow meter M100 Smart‐Track (Sierra Instruments) which was calibrated with Air (NL min^−1^). Qualitative analysis of the gas mixture was done by a 4‐channel micro‐GC (μ GC, model 4900) from Agilent Technologies (H_2_, O_2_, N_2_, CO, CO_2_, C1‐C5, MeOH, H_2_O). The gas flow was corrected with correction values given by the manufacturer of the mass‐flow meter. For the correction, only N_2_, CO_2_, H_2_, and O_2_ were considered. Temperature and pressure were detected to calculate the mass‐flow in L min^−1^.

For analysis of the organic phase, the phase was harvested from the aqueous reaction mixture after the phases had fully separated. It was diluted in n‐pentane and measured with a gas chromatograph (Agilent Technologies, model: 7980B, coupled with a 5977 A mass selective detector) equipped with a polysiloxan column (Agilent Technologies, CP9092, 0.1 μm film, VF‐5ht UltiMEtal), auto‐sampler (model: 7693) and MMI‐injector. Injection volume was 1 μL with a split ratio of 5 : 1 and 320 °C injector temperature. The oven was set to 35 °C for 6 min and increased to 275 °C applying a ramp of 15 K min^−1^ and 1 min dwell time at this temperature. The mass spectrometric data were acquired and processed using the GC‐MS (gas chromatography‐mass spectrometry) data system (MassHunter). Peaks were identified and assigned based on comparison with NIST‐mass spectra library. For the quantification using the FID, all peak areas were integrated and corrected with the molar response factor (referred to n‐decane) that has been previously determined with standards. Selectivity of the products was than calculated from corrected peak areas. Measurement error was based on standard deviation of sample measurements (four times per sample) and error of determined correction factor.

The aqueous phase was analyzed using Raman spectroscopy, undiluted and without any further treatment. Raman measurements were conducted using a HyperFlux Pro Plus 785 (Tornado Spectral Systems) with 785 nm laser wavelength and a laser power output of 495 mW. Exposure time for each spectrum was 1 s and 10 measurements were averaged to one spectrum. The laser system was controlled with the software Tornado Spectral Soft. Spectra were processed with panorama 4.0 (LabCognition, Analytical Software GmbH & Co. KG). Concentrations of carboxylic acid were determined based on a five‐point external standard calibration. For each sample, a minimum of three spectra was taken and measurement error is based on standard deviations.

Energy dispersive X‐ray (EDX) measurements were carried out on an Oxford Instruments INCA II using accelerating voltage of 10 kV.

The pH value of the starting solution and aqueous reaction mixture were determined using a pH 330i (WTW GmbH) with a basic pH combination electrode (gel reference system, SenTix®41) with temperature sensor. Calibration was carried out according to DIN 19266 (1.679/4.006/6.865).

### Calculation

Conversion *θ* of the collected aqueous phase and the aqueous phase taken after the reactor via the T piece is calculated according to Equation 1 where *c*_0_ is the starting concentration of carboxylic acid and *c*_x(t)_ the concentration after electrolysis.

(1) $$\theta =\frac{c_{0}-c_{x\left(t\right)}}{c_{0}}\cdot 100$$

Calculation of the Faraday Efficiency (FE) for the detected product *p* is done by the ratio of charge required to build the amount of product and the applied charge according to current *I* and residence time in the reactor *τ* [Eq. 2]. The number of transferred electrons *z*, the concentration of the product *c*_p_, reactor volume *V*_R_ and Faraday constant *F* calculates the numerator of the equation. A table with the number of transferred electrons depending on the product can be found in the Supporting Information.

(2) $${FE}_{P}\left[\%\right]=\frac{z\cdot c_{P}\cdot V_{R}\cdot F}{I\cdot \tau }\cdot 100$$

Concentration of the product was calculated using conversion *θ*, selectivity of the product *σ_P_*, starting concentration of carboxylic acid *c*_0_ and a stoichiometric factor *n* depending on the product [Eq. 3].

(3) $$c_{P}=n\cdot \theta \cdot \sigma_{P}\cdot c_{0}$$

In order to get the Faraday Equivalents (FEq.) applied per residence time Equation (2) is inverted and the beginning concentration of the carboxylic acid *c*_0_ is used instead of the product concentration. Number of transferred electrons is selected to be *z*=1 considering only the first oxidation step to get alkyl radicals [Eq. 4].

(4) $$FEq=\frac{I\cdot \tau }{c_{0}\cdot V_{R}\cdot F\cdot z}$$

The residence time *t*_R_ is calculated according to the volume of the electrochemical cell *V_cell_* which is given by the dimensions of the microchannels and the volumetric flow rate of the electrolyte *Q* [Eq. 5].

(5) $$t_{R}=\frac{V_{cell}}{Q}$$

## Conflict of interest

The authors declare no conflict of interest.

1

## Supporting information

As a service to our authors and readers, this journal provides supporting information supplied by the authors. Such materials are peer reviewed and may be re‐organized for online delivery, but are not copy‐edited or typeset. Technical support issues arising from supporting information (other than missing files) should be addressed to the authors.

Supporting Information

## Data Availability

The data that support the findings of this study are available from the corresponding author upon reasonable request.
