# Supplementary material for: Scalable Microreactor Concept for the Continuous Kolbe Electrolysis of Carboxylic Acids Using Aqueous Electrolyte
Source: ChemistryOpen. 2022 Oct 6;11(10):e202200171. doi: 10.1002/open.202200171 (PMC9535501; doi:10.1002/open.202200171)
Supplement: Supplementary file 1 — Supporting Information [file OPEN-11-e202200171-s001.pdf]

ChemistryOpen

Supporting Information

Scalable Microreactor Concept for the Continuous Kolbe Electrolysis of Carboxylic Acids Using Aqueous Electrolyte

Nils Baumgarten,* Bastian J. M. Etzold, Juri Magomajew, and Athanassios Ziogas*

Supporting Information

Content

1. Selectivity of Side Products in Current Density and Residence Time Variation 3
2. Productivity and Energy Consumption 4
3. Energy Dispersive X-Ray Measurements...5
4. Electrochemical Microreactor and Experimental Setup...6

1. Selectivity of Side Products in Current Density and Residence Time Variation

In Figure S1 selectivity for the main detected side products heptanal, branched hepten originating from β-H-elimination (non-Kolbe pathway) and ester (heptyl-octanoate) that was observed in current density as well was flow rate variation is depicted. For the first variation lowest amount of side products was found for 0.28 A cm^{-2} and 0.48 A cm^{-2}. Considering residence time variation a clear trend is only detectable for heptanal where the amount is increasing when higher residence times are applied. For carboxylic ester a slightly decrease with increasing residence time was found whereas the amount of hepten shows no real dependency on the residence time.

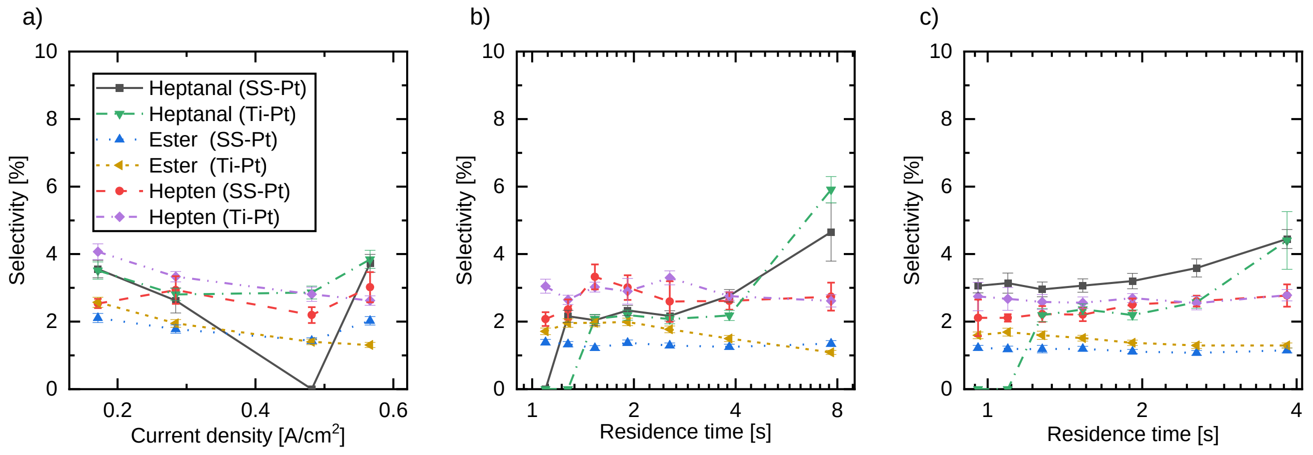

Figure S1. Selectivity for side products heptanal, hepten (non-Kolbe) and carboxylic ester (heptyl-octanoate) depending on current densities (a) and residence time applying 0.28 A cm^{-2} (b) and 0.48 A cm^{-2} (c). Abbreviations in brackets mark the different anode materials used in the electrolysis. (SS-Pt: platinized stainless steel, Ti-Pt: platinized titanium).

In addition to side product selectivity Figure 2 shows a magnified extract of the FE for the OER when different current densities and residence times were applied. The discussion was already done in the main article. According to table S1, different amounts of transferred electrons were considered for the FE calculation of every detected product:

Table S1. Number of transferred electrons depending on the detected product for FE calculation (according to equation 2).

Product / compound	Number of transferred electrons
Heptane	1
1-Hepten	1
Tetradecane	2
Heptanal	4
1-Heptanol	2
Ester (heptyl-octanoate)	2
Hepten (non-Kolbe)	2
HER (H_2)	2
OER (O_2)	4

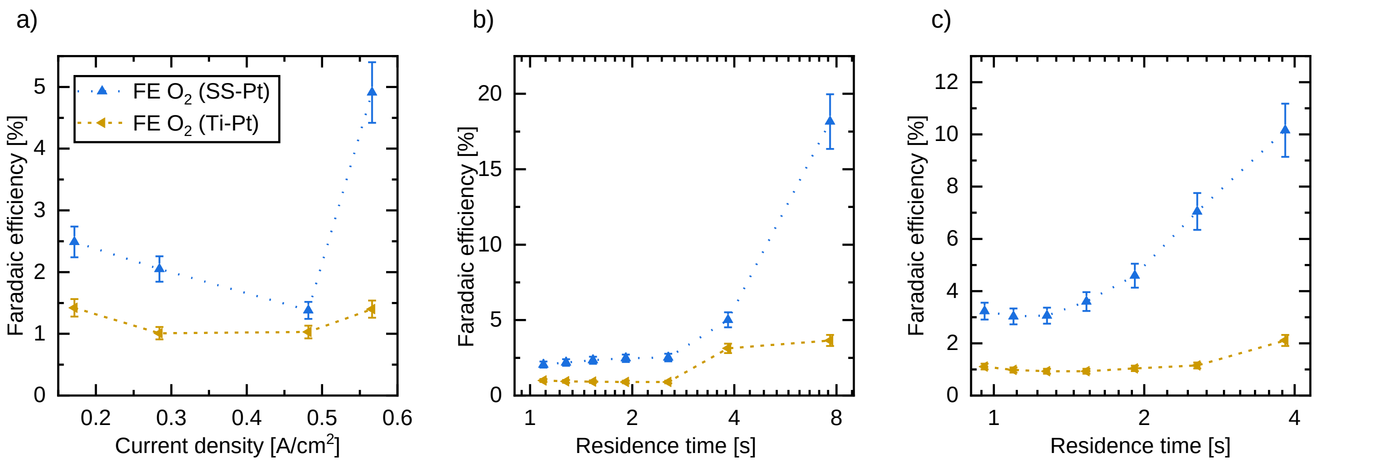

Figure S2. Zoom-in for faradaic efficiency for the oxygen evolution reaction in relation to different current densities (a, residence time: 2.55 s) and flow rates at 0.28 A cm^{-2} (b) and 0.48 A cm^{-2} (c). Blue data points were generated with the SS-Pt || Ni-SS setup and yellow data points using the Ti-Pt || Pt-SS setup.

Apart from the organic side products also the appearance of the aqueous solution after the electrolysis changed from colorless to light yellow increasing residence time when platinized stainless steel was used as anode material. The color can be assumed to originate from iron oxide formation as corrosion effect on the working electrode. For platinized titanium as anode material no effect was visible.

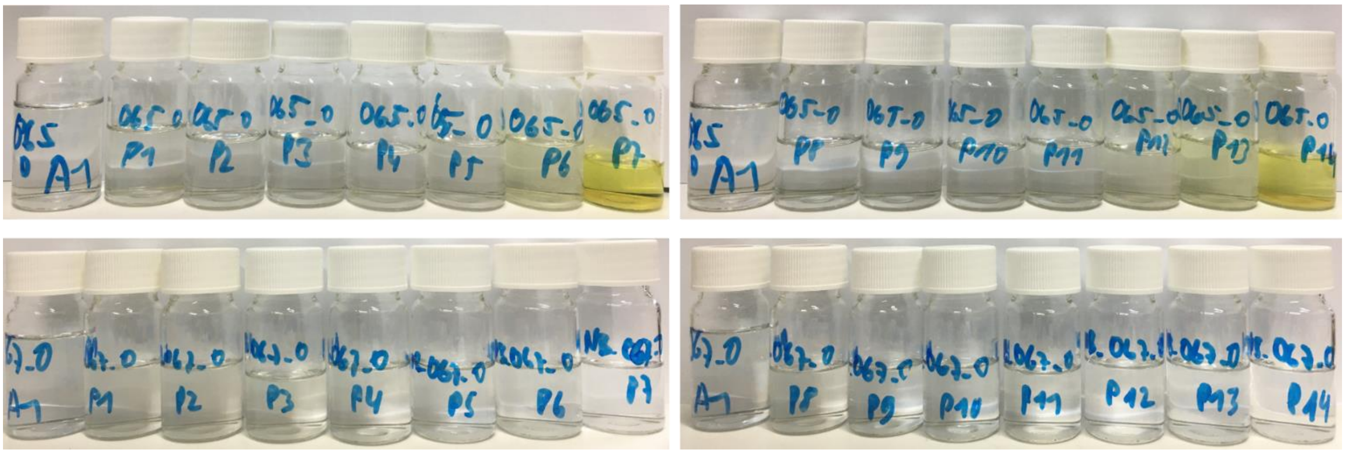

Figure S3. Picture of aqueous phase reaction mixture after electrolysis and fully phase separation for different residence times and current densities using SS-Pt || Ni-SS setup (upper) and Ti-Pt || Pt-SS setup (bottom). Left: 0.28 A cm^{-2} at residence time range from 1.1 s to 7.7 s. Right: 0.48 A cm^{-2} at residence time range from 0.96 s to 3.8 s.

2. Productivity and Energy Consumption

For residence time variation productivity for the main Kolbe products in mol h^{-1} and energy consumption in kWh to synthesize one mole was calculated according to following equations where Q is the overall flow rate and c_P the concentration of the product. Energy consumption in kWh mol^{-1} was calculated by the FE of the product, cell voltage U_{cell}, Faraday constant F (in kAh mol^{-1}), the number of transferred electrons z.

Except the local maximum at 2.55 s residence time (0.28 A cm^{-2}) a clear dependency on the calculated values can be seen where low residence time results in higher productivity and lower energy consumption. Regarding a continuous process it can be assumed to be more effective applying short residence times resulting in increased performance parameters for the electrochemical reaction.

$$Productivity[\mathrm{mol\ h^{-1}}] = Q \cdot c_P \quad \text{(S1)}$$

$$Energy\ consumption[\mathrm{kWh\ mol^{-1}}] = \frac{z \cdot F \cdot U_{cell}}{FE} \quad \text{(S2)}$$

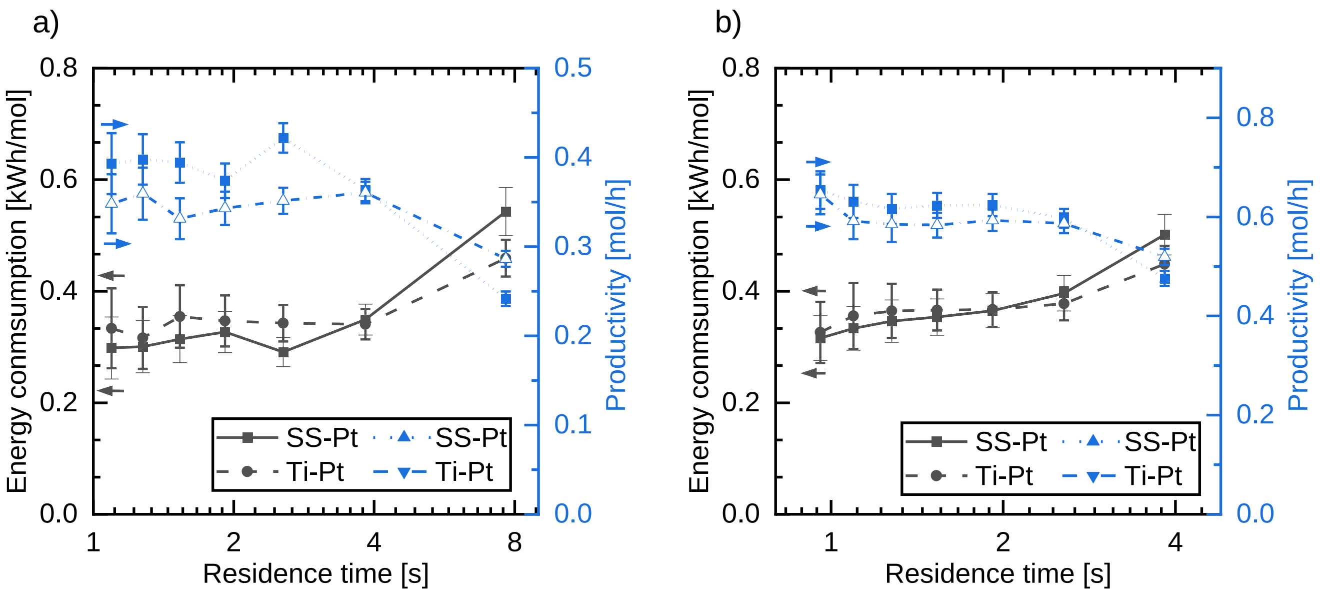

Figure S4. Productivity for Kolbe products tetradecane, heptane and 1-hepten (right axis) and energy consumption (left axis) depending on residence time at current densities of 0.28 A cm^{-2} (a) and 0.48 A cm^{-2} (b). Abbreviations in brackets mark the different anode materials used in the electrolysis. (SS-Pt: platinized stainless steel, Ti-Pt: platinized titanium).

3. Energy Dispersive X-Ray Measurements

In order to analyze the composition of the layer on the electrodes and the isolated powder from electrodes and reaction mixture, energy dispersive X-Ray (EDX) measurements were carried out. Results of the atomic percentage of detected elements can be seen in Table S2. In all there cases, where powder was isolated either from the electrodes or from the reaction mixture, iron and oxygen were found as main elements, assuming that iron oxide was built during the reaction when long electrolysis times are or high current densities in combination with high residence times were applied. Because of mechanical stress or incomplete electroplating of Pt at geometrically hindered areas, the stainless steel parent material is corroding. In addition, the part between HPLC tubing connection of the 3-D printed electrode and inlet of the electrolyte there is a small inner part which is neither electroplated with Pt nor coated with insulating PTFE. Accordingly, corrosion can also take place in this small area and built iron oxide is flushed out of the reactor and plated on the cathode. Also carbon was found in large amounts, however most of the detected quantities can be dedicated to contamination or originates from the measurement underground. Certainly, it would also be possible that carbon could come from (over-oxidized) carboxylic acid residues, but the layer on the electrodes appears more like tarnishing and brittleness effects that could originate from surface interactions with built hydrogen.
In addition to the isolated powders also different areas on the counter electrode were analyzed using EDX. For inactive or visibly clean areas mainly Ni was detected in addition to some minor amount of oxygen and carbon. Comparable results were obtained for the spacer area located between the reaction channels. For active counter electrode areas the amount of detected iron and oxygen was increasing manifesting that iron oxide is deposited on the counter electrode. Also carbon content is increasing originating from reaction mixture (carboxylic acid) or product (hydrocarbon) residues.

Table S2. Atomic percentage of elements found in EDX analysis of electrodes and isolated powder.

	Inactive cathode[a]	Active cathode[a]	Anode powder[b]	Cathode powder[c]	Powder from reaction mixture[d]
Element	Atomic percent [%]	Atomic percent [%]	Atomic percent [%]	Atomic percent [%]	Atomic percent [%]
C	8.7 ± 0.4	24 ± 1	46 ± 5	36 ± 3	20 ± 5
O	11.2 ± 0.6	36 ± 2	39± 3	43± 1	52 ± 5
Fe	2.3 ± 0.1	24 ± 1	6.2 ± 0.8	13.5 ± 0.2	20.1 ± 0.9
Ni	77.0 ± 4	14 ± 3	0.4 ± 0.2	2.7 ± 0.2	3.6 ± 0.7
K	0.75 ± 0.04	1.1 ± 0.2	8 ± 1	2.1 ± 0.1	3.5 ± 0.5
Cr	-	1.5 ± 0.2	-	-	-
Si	-	-	0.33 ± 0.04	0.6 ± 0.1	0.39 ± 0.06

[a] Inactive (spacer, verge) and active parts of nickel coated stainless steel cathode after electrolysis. [b] Red-brown powder isolated from the anode (platinized stainless steel) surface after cell was disassembled. [c] Solid residue that was deposited on the nickel coated stainless steel cathodes. Isolation was done via sonication of the electrode in isopropanol and evaporation of the solvent. [d] Red-brown precipitation in reaction mixture isolated via filtration.

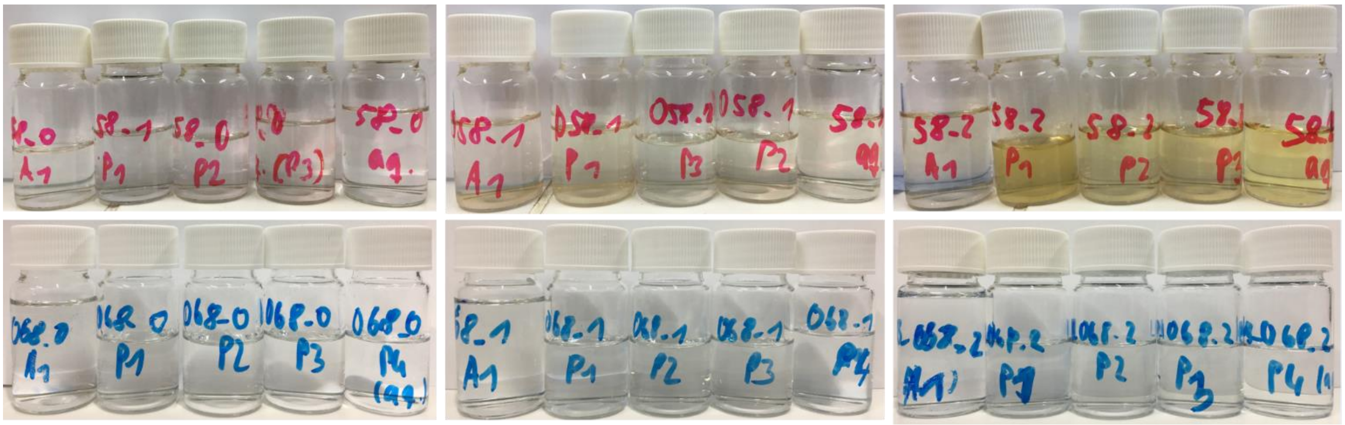

Figure S5. Picture of samples of aqueous starting solution and aqueous reaction mixture after electrolysis and full phase separation. Upper pictures show samples when SS-Pt || Ni-SS was used as reactor setup where aqueous phase is turning from colourless to light-yellow/orange over the three cycles. Pictures on bottom show samples when Ti-Pt || Pt-SS setup was used over all three cycles (from left to right). No visible color-change was detectable.

4. Electrochemical Microreactor and Experimental Setup

The reactor was operated with two cells in parallel with a different electrode setup: 1) SS-Pt || Ni-SS-Ni || Pt-SS 2) SS-Pt || Pt-Ti-Pt || Pt-SS. A HPLC pump was used to pump the reaction medium through the electrochemical cells and a manifold just before the cells allows the parallel feed of reaction medium in both cells. Dimensions of the microstructured electrodes were 100×0.76×0.15 mm^3 including 56 microchannels per electrode side. Reaction volume of an undivided cell was 0.64 mL with an active electrode area of 42.6 cm^2. The cells were assembled in an iron press and Chemraz® seals were used to prevent leakage. Figure S5 shows the experimental setup with two microstructured end electrodes and a plane electrode in between assembled in the iron press housing. In addition a picture of a platinized, microstructured electrode is depicted.

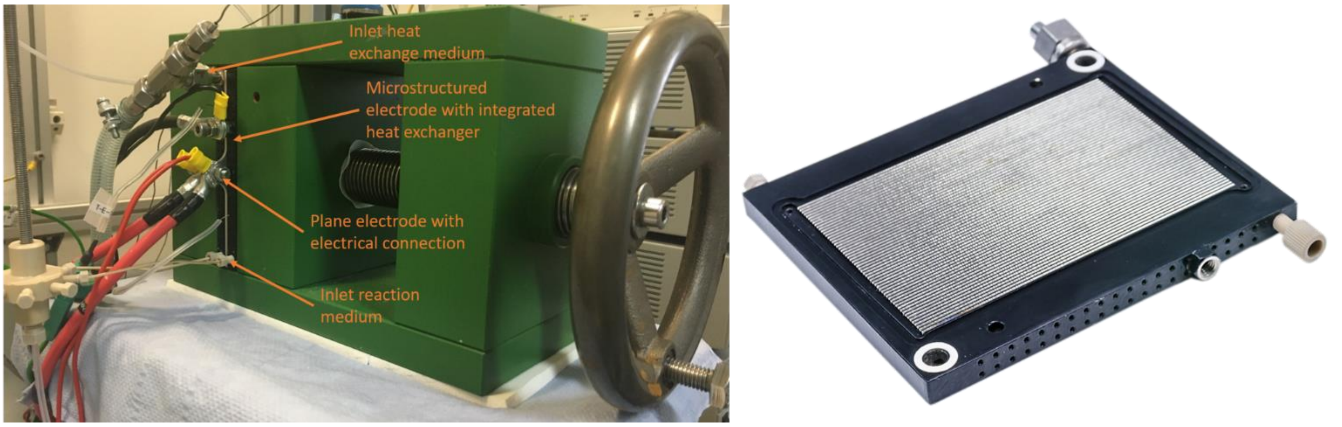

Figure S5. Picture of a two cell setup on the left. Two microstructured electrodes and a plane electrode in between are assembled in the iron press allowing the operation of two cells in parallel. Right picture shows a platinum coated microstructured electrode (picture: Tobias Hang, Fraunhofer IMM).
